# Two new species of the genus *Metapocyrtus* Heller, 1912 (Coleoptera, Curculionidae, Entiminae, Pachyrhynchini), subgenus *Orthocyrtus* Heller, 1912, from Mindanao Island, Philippines

**DOI:** 10.3897/zookeys.1029.63023

**Published:** 2021-04-08

**Authors:** Analyn A. Cabras, Milton Norman Medina, Maurizio Bollino

**Affiliations:** 1 Coleoptera Research Center, Institute of Biodiversity and Environment, University of Mindanao, Davao City, 8000, Philippines University of Mindanao Davao City Philippines; 2 Museo di Storia naturale del Salento, 73021 Calimera, Lecce, Italy Museo di Storia naturale del Salento Calimera Italy

**Keywords:** Bionomic, new taxa, taxonomy, urban biodiversity, weevils

## Abstract

Two new species of *Metapocyrtus* Heller, 1912, subgenus *Orthocyrtus* Heller, 1912 (Coleoptera, Curculionidae, Entiminae, Pachyrhynchini) are described and illustrated from Mindanao Island, Philippines. The species are Metapocyrtus (Orthocyrtus) davaoensis**sp. nov.** and Metapocyrtus (Orthocyrtus) hirakui**sp. nov.** from Davao City and Bukidnon, respectively. Brief bionomical notes and phenotypic characters compared to their sympatric Entiminae counterparts are also reported. The discovery of M. (O.) davaoensis**sp. nov.** in Davao City confirms how understudied Coleoptera are in Mindanao and underlines the potential for the discovery of new species even in highly urbanized areas.

## Introduction

The tribe Pachyrhynchini has achieved notoriety for its beautiful iridescent markings making its species conspicuous and one of the most well-known beetle taxa in the Philippines. It currently counts 18 genera with roughly 500+ described species. Being wingless, most Pachyrhynchini have a very narrow geographic range and are mostly endemic to an island or a mountain range. According to the Forest Management Bureau of the Department of Environment and Natural Resources, forest cover in the Philippines has dropped by 70%, from 21 million hectares in 1900 to approximately 6.5 million hectares in 2007. This loss of forests has consequently caused the majority of the species from this tribe to be listed as Vulnerable in the latest national assessment of the Department of Environment and Natural Resources (DENR Administrative Order 2019). The genus *Metapocyrtus* Heller, 1912 is endemic in the Philippines and is the most speciose and complex within the tribe (Schultze 1923; Cabras et al. 2018; Bollino et al. 2020). Metapocyrtus subgenus Orthocyrtus Heller, 1912 currently counts more than 36 species distributed all over the Philippines: 18 species were described from Luzon, four species and one subspecies from Visayas, 12 species from Mindanao Island, and three were generically described from the Philippines (Schultze 1925; Cabras et al. 2018; Cabras and Medina 2019).

Mindanao Island is the second largest island in the Philippines with a total area of 97,530 km^2^ divided into six political regions and five faunal biogeographic subregions. The unique biogeographic history of Mindanao Island was formed as a by-product of paleo-island accretion in identified suture zones (Yumul 2003), and its recent connectivity to several shallow-water neighboring islands during the last Pleistocene sea-level fluctuations (e.g., Samar, Leyte, Bohol, Dinagat, and Siargao). Such inter-island connectivity led to the formation of the identified Greater Mindanao Pleistocene Aggregate Island Complex which allowed for faunal exchange and gene flow among species (Rohling et al. 1998; Brown and Diesmos 2001). Despite the recent efforts to document the island’s coleopterological fauna, many species remained unclassified including two new taxa recently collected in Davao City and Bukidnon. Upon examination, the new species have been found to belong to the subgenus Orthocyrtus based on characters indicated by Cabras et al. (2018). Both species and their habitats are described and illustrated here; we also include some comments on sympatric beetles which apparently belong to the same mimetic ring.

## Materials and methods

The specimens deposited in the University of Mindanao Coleoptera Research Center were collected through sheet beating and hand picking and killed in vials with ethyl acetate. Morphological characters were observed under Luxeo 4D and Nikon SMZ745T stereomicroscopes. The illustrations, as well as the treatment of the genitals, were identical to those described by Bollino and Sandel (2017), including that of endophallus eversion by using the Berti-Vachon method (Bontems 2013). Images of the habitus and genitalia were taken using a Nikon D5300 digital camera with a Sigma 18–250 macro lens, while the image of the endophallus was taken with a Nikon D90 digital camera, and Laowa 25 mm f/2.8 2.5–5X Ultra Macro lens. All images were stacked and processed using a licensed version of Helicon Focus 6.7.0 and Photoshop CS6 Portable software. Label data are indicated verbatim. Measurements mentioned in this paper are abbreviated as follows:

/ different lines

// different labels

**â** arithmetic mean rounded to one decimal place;

**LB** body length, from the apical margin of pronotum to the apex of elytra;

**LE** elytral length, from the level of the basal margins to the apex of elytra;

**LP** pronotal length, from the base to apex along the midline;

**LR** length of rostrum;

**WR** maximum width across the rostrum;

**WE** maximum width across the elytra;

**WP** maximum width across the pronotum.

Comparative materials and specimens used in the study are deposited in the following institutional collections:

**DUBC** Daugavpils University Beetle Collection, Daugavpils, Latvia;

**MBLI** private collection of Maurizio Bollino, Lecce, Italy;

**NIAES**National Institute for Agro-Environmental Sciences, Tsukuba, Japan;

**SMTD** Senckenberg Natural History Collections, Dresden, Germany;

**UMCRC** University of Mindanao Coleoptera Research Center, Davao City Philippines;

**ZMPC** private collection of Zhao Ming, Guangzhou, China.

## Results

### Metapocyrtus (Orthocyrtus) davaoensis sp. nov.

Taxon classificationAnimaliaColeopteraCurculionidae

F8C1665F-2E96-59C4-8527-23B67B4B2FB6

http://zoobank.org/DC3785AE-085A-4E26-8AA8-460601F0DA9D

Figs 1 , 2 , 3 , 4

#### Holotype

**Figure 1.**
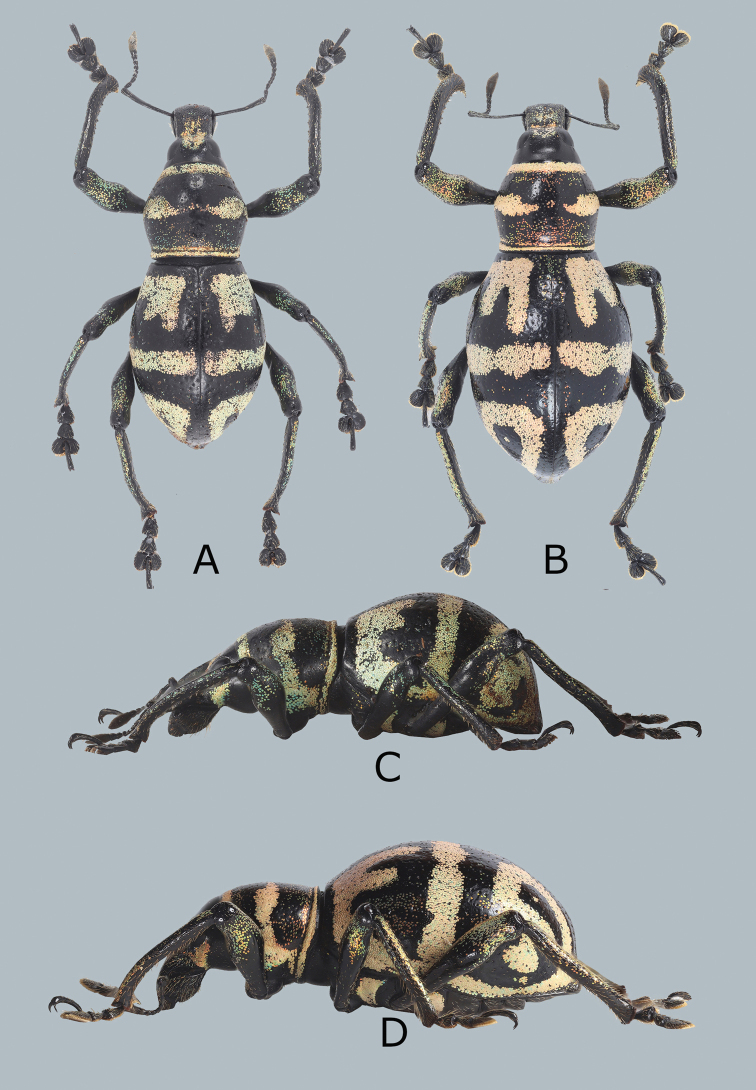
Metapocyrtus (Orthocyrtus) davaoensis sp. nov. **A** male holotype, dorsal view **B** female, dorsal view **C** ditto,male, lateral view **D** ditto,female, lateral view.

(Figs 1, 3), male: Philippines – Mindanao / Calinan / Davao City / March.2018 / coll. Medina (typed on white card) // HOLOTYPE male / Metapocyrtus (Orthocyrtus) davaoensis / CABRAS, MEDINA & BOLLINO, 2021 (typed on red card). Presently in UMCRC, will be deposited in National Museum of Natural History (PNMNH) under the National Museum of the Philippines.

#### Paratypes

(4♂♂, 6♀♀): 1♂, 4♀♀, Philippines – Calinan, Davao City, Mindanao Island / March 2018/ coll. Cabras, all in UMCRC; 1♂, 1♀, Philippines – Gumitan, Davao del Sur, Mindanao Island/ XI, 2019/ Leg-local collector/, in coll. ZMPC; 1♂, Philippines – Mindanao I. / Tabayon – Davao del Sur / March 2014 / Lg. local people - coll. Bollino; 1♂, Philippines – Mindanao / Kapatagan / (Davao del Sur) / January 2016 / Lg. local people – coll. Bollino; 1♀, Philippines – Mindanao / Cabanglasan – Bukidnon / XII.2014–I.2015 / ex Lumawig - coll. Bollino, all in MBLI. All paratypes with additional red label: Paratype / Metapocyrtus (Orthocyrtus) davaoensis / CABRAS, MEDINA, & BOLLINO, 2021.

#### Diagnosis.

Metapocyrtus (Orthocyrtus) davaoensis sp. nov. in general appearance resembles Metapocyrtus (Orthocyrtus) mansaka Cabras, Bollino & Medina, 2018 from Davao de Oro, South Cotabato, and Agusan del Sur, but differs for its unique elytral ornamentation, pronotal markings and shape of the aedeagus. Metapocyrtus (O.) davaoensis elytral ornamentation consist of short stripes from behind the base to before the middle of stria II and behind the base to the basal third of interval IV, a thin median transverse band starting from interval I towards the lateral margin, a distorted subtriangular stripe on apical third extending from stria I to interval IV, and a long stripe from behind the base to the apex along the lateral margin, confluent with the basal, medial and apical stripes. Metapocyrtus (O.) mansaka has three broad, scaly bands on the elytra; for pronotal marks, M. (O.) davaoensis has thin, transverse, scaly marks along the entire width in the middle compared with M. (O.) mansaka which has triangular transverse scaly marks from each side dilated towards the middle but not confluent.

#### Description.

**Male.** Dimensions (in mm): LB 10.0–10.3 (holotype 10.0, â: 10.15), LR 1.8–1.9 m (1.8, â: 1.85), WR 1.5–1.6 (1.5 mm, â: 1.55), LP 3.0–3.3 (3.0, â: 3.15), WP: 3.3–3.4 (3.3, â: 3.35), LE 6.4–6.6 (6.4, â: 6.5). WE 4.7–4.9 (4.7 mm, â: 4.8). *N* = 3.

Integument black. Body surface, rostrum, head, and underside with a weak luster.

***Body*** subglabrous. ***Head*** glabrous, sparsely minutely pubescent on ventral side, with metallic, pale-yellow ochre, elliptical scales on each side and turquoise, hair-like scales on lateroventral parts; forehead between eyes covered with metallic, light-yellow ochre, round scales. ***Rostrum*** strongly rugose, longer than wide (LR/WR: 1.2), bearing minute, yellow-ochre, adpressed hairs on the lateral surface, and lateroventral sides below antennal scrobe covered with long, light-colored hairs; transverse basal groove distinct; longitudinal groove along midline distinct, creating a shallow depression beset with metallic, golden-yellow, round scales; lateral sides with round to elliptical light-yellow ochre scales; dorsum finely punctured; dorsal surface weakly convex. Eyes medium-sized and feebly convex. Antennal scape and funicle of almost the same length, moderately covered with fine, light-colored hairs. Funicular segments I and II almost of the same length, twice as long as wide; segments III–VII nearly as long as wide; club subellipsoidal, nearly three times longer than wide. ***Prothorax*** subglobular, slightly wider than long (LP/WP: 0.91), finely punctured, widest at middle, weakly convex, sparsely covered with turquoise and light-yellow ochre, round scales on basal half of dorsum, and with the following scaly markings of metallic, light-yellow ochre and turquoise, round scales: a) thin band at the anterior margin, b) transverse band in the entire width in middle, c) thin band at the posterior margin, and d) broad lateroventral stripe before the coxa confluent with the anterior and posterior marginal bands. ***Elytra*** subovate (LE/WE:1.36), slightly wider and moderately longer than prothorax (WE/WP: 1.42, LE/LP: 2.13), body surface black with sparse, golden-yellow and turquoise, round scales, subglabrous, finely punctured, moderately convex; apex with sparse, white, fine hairs. Each elytron with the following scaly markings of metallic, light-yellow ochre to turquoise, round scales: a) short stripe from behind base to before middle of stria II, b) short stripe from behind base to basal third of interval IV, basally confluent with stripe on stria II, c) thin, median, transverse band starting from interval I towards lateral margin, d) distorted subtriangular stripe on apical third extending from stria I to interval IV, e) small dot on interval V at apical quarter, at times confluent with the distorted subtriangular stripe, f) long stripe from behind base to apex along lateral margin, confluent with basal, median, and apical stripes. ***Legs*** with strong clavate femora. Femora sparsely covered with light-colored hairs, with apical half covered with turquoise, elliptical scales. Tibiae covered with subrecumbent, light-colored bristles and metallic-turquoise and golden-yellow elliptical scales towards apical part, weakly serrate along inner edge. Fore tibiae bear a mucro at apex. Tarsomeres covered with sparse pubescence. Coxae barely pubescent with very sparse, turquoise, hair-like scales. Mesosternum covered with light-colored, adpressed bristles. Metasternum with light-colored, adpressed bristles and sparse, light-yellow ochre, round scales at lateral sides. Ventrite 1 depressed on disc, with light-yellow ochre and turquoise, round scales towards lateral margin. Ventrites 2–5 sparsely covered with adpressed bristles, especially towards margin. Ventrite V flattened, apical half finely densely punctured, interspersed sparsely with bluish, hair-like scales. Ventrites 1–5 with dense and long, light-brown bristles, laterally with sparse light-green bristles.

**Figure 2.**
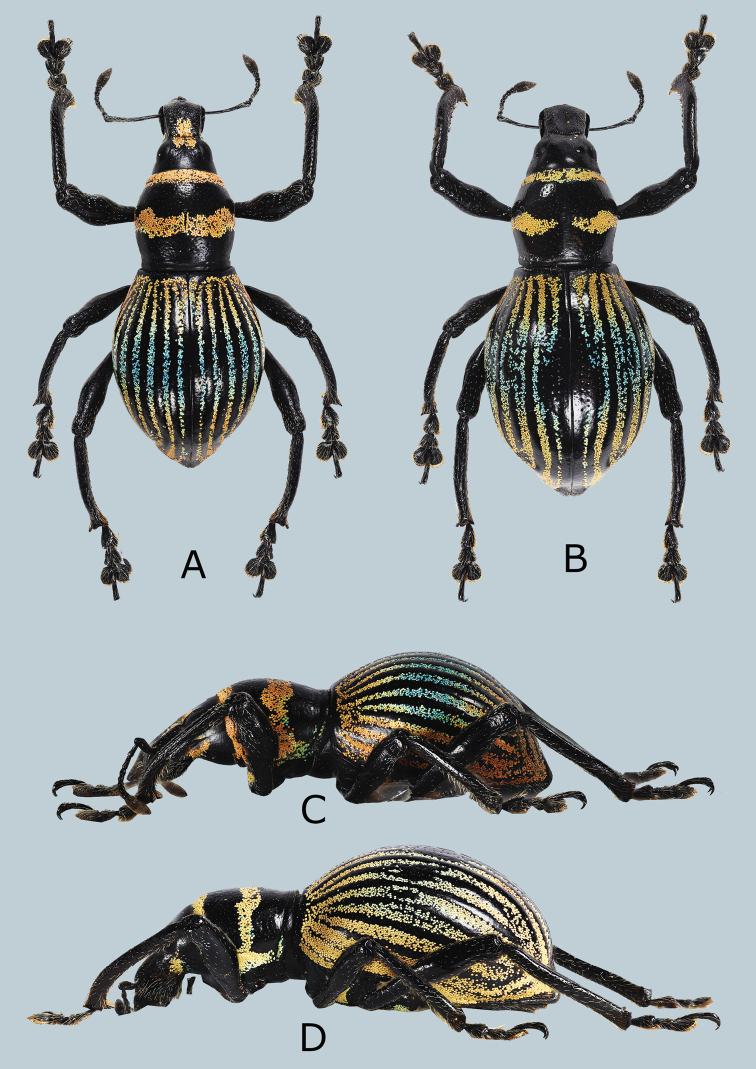
Metapocyrtus (Orthocyrtus) hirakui sp. nov. **A** male holotype, dorsal view **B** female, dorsal view **C** ditto, male, lateral view **D** ditto, female, lateral view.

***Male genitalia*** as shown in Figure 2A–C.

Due to of the few males available (only three, excluding the holotype), we were unable to obtain even a partial evertion of the endophallus (read below for more comments).

**Female.** Dimensions (in mm): LB 12.0–12.7 (â: 12.33), LR 2.0.–2.1 (â: 2.05), WR 1.7–1.8 (â: 1.75). LP 3.0–3.2 (â: 3.08). WP 4.2–4.5 (â: 4.33), LE 9.0–9.5 (â: 9.23). WE 6.9–7.5 (â: 7.18). *N* = 4.

Habitus as shown in Figure 1D–F.

Females differ from males in the following: a) pronotum wider than long (LP/WP 0.71), slightly shorter than in male; b) pronotum imperfectly subglobular, and c) elytra imperfectly subovate (LE/WE 1.27–1.3), longer and wider (WE/WP 1.64–1.67, LE/LP 2.97–3.0) than in male, widest before middle; d) ventrite 1 flattened or slightly convex on disc. Otherwise female similar to the male.

#### Etymology.

The new species is named after its type locality, Davao City.

#### Distribution.

Metapocyrtus (Orthocyrtus) davaoensis sp. nov. is known from Calinan, Davao City and Gumitan, Davo del Sur, and Cabanglasan, Bukidnon.

**Figure 3.**
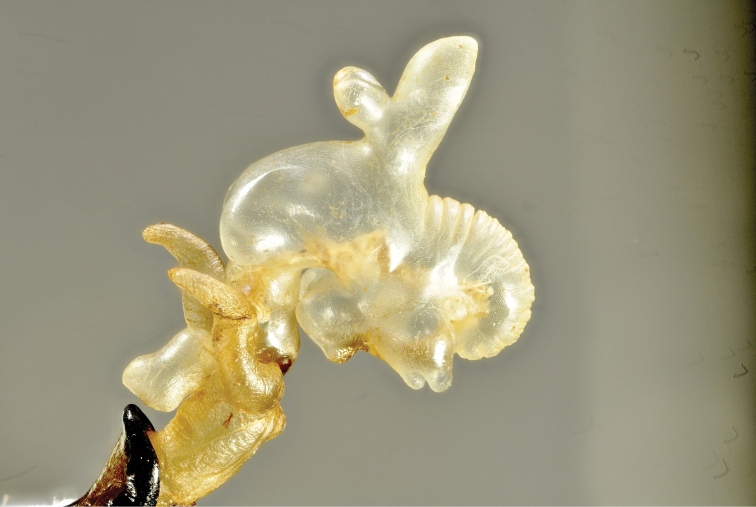
Metapocyrtus (Orthocyrtus) hirakui sp. nov. everted endophallus, lateral view.

**Figure 4.**
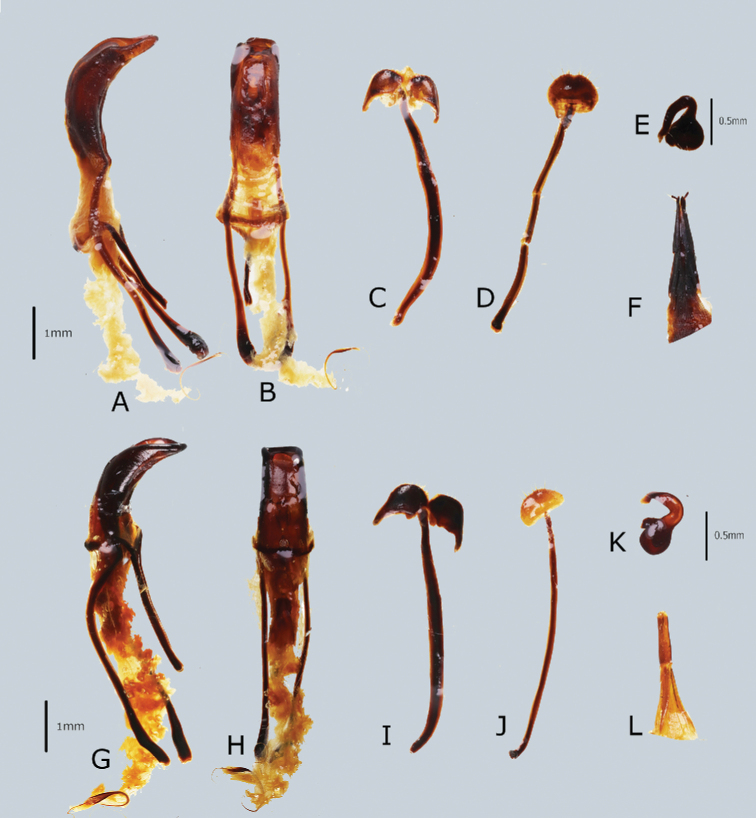
Male genitalia and female terminalia of *Orthocyrtus* spp. **A–F**Metapocyrtus (Orthocyrtus) davaoensis sp. nov. **A** penis in lateral view **B** idem. in dorsal view **C** sternite IX in dorsal view **D** sternite VIII in ventral view **E** spermatheca **F** ovipositor in dorsal view **G–L**Metapocyrtus (Orthocyrtus) hirakui sp. nov. **G** penis in lateral view **H** idem. in dorsal view **I** sternite IX in dorsal view **J** sternite VIII in ventral view **K** spermatheca **L** ovipositor in dorsal view.

### Metapocyrtus (Orthocyrtus) hirakui sp. nov.

Taxon classificationAnimaliaColeopteraCurculionidae

5F165537-2E48-5E75-90A7-3C324E9AEC17

http://zoobank.org/B6BFAE9D-74BD-46B5-B6FF-C66AAE39BFFD

Figs 5 , 6

#### Holotype

(Fig. 1A, B), male: Philippines – Mindanao / Bukidnon / Lantapan / July 2018 / coll. Medina (typed on white card) // HOLOTYPE male / Metapocyrtus (Orthocyrtus) hirakui / CABRAS, MEDINA & BOLLINO, 2021 (typed on red card) Presently in UMCRC, it will be deposited in Philippine National Museum of Natural History (PNMNH) under the National Museum of the Philippines (NMP).

#### Paratypes.

28♂♂, 39 ♀♀: 3♂♂, 15 ♀♀, Philippines – Mindanao/ Bukidnon / Lantapan / V–VII.2018 / coll. Medina; 7♂♂, 4 ♀♀, Philippines – Mindanao/ Bukidnon/ Talakag/ VII. 2018/ Leg, L.C. All in UMCRC; 7♂♂, 2♀♀: Philippines – Mindanao / Cabanglasan – Bukidnon / III–IV.2013 / m. 800–1000 – Leg. Loc. people / coll. M. Bollino; 7♂♂, 14♀♀: Philippines – Mindanao / Cabanglasan (Bukidnon) / September 2013 / ex. N. Mohagan / Lg. local people – coll. Bollino; 1♂, 1♀: Philippines – Mindanao / Cabanglasan / (Bukidnon) / December 2018 – January 2019 / Lgt. local people – coll. Bollino; 1♂: Philippines – Mindanao / Kalatungan / (Bukidnon) / X.2015 / ex I.Lumawig – coll. Bollino; 2♂♂, 3 ♀♀: Philippines – Mindanao / San Fernando – Bukidnon / X.2013 / ex Lumawig / lg. Local people – coll. Bollino, all in MBLI. All paratypes with additional red label: PARATYPE / Metapocyrtus (Orthocyrtus) hirakui / CABRAS, MEDINA & BOLLINO, 2021

#### Diagnosis.

Metapocyrtus (Orthocyrtus) hirakui sp. nov. differs from all other species of the subgenus for its unique elytral ornamentation consisting of yellow ochre to blue longitudinal stripes and striae I to II occasionally interrupted in the middle, although generally such striae are continuous from the base to the apex of elytra.

#### Description.

**Male.** Dimensions (in mm): LB 10.6–10.9 (holotype 10.9, â: 10.75) . LR: 1.9–2.0 (holotype 2.0, â: 1.95), WR 1.3–1.5 (holotype 1.5, â: 1.4), LP: 3.0–3.1 (holotype 3.0, â: 3.05), WP 3.0–3.1 (holotype 3.0, â: 3.05), LE: 6.9–7.0 (holotype 6.9, â: 6.95), WE: 4.9–5.0 (holotype 5.0, â: 4.95). *N* = 4.

Integument black. Body surface, rostrum, head, and underside subopaque.

***Body*** subglabrous. ***Head*** subglabrous, sparsely, minutely pubescent, with light-yellow ochre, round scales interspersed with metallic-white, hair-like, elliptical scales on lateroventral parts; forehead between eyes partially covered with metallic, light-yellow ochre, round scales; median groove barely distinct, not reaching the vertex. ***Rostrum*** sparsely, minutely pubescent, slightly longer than wide (LR/WR: 1.33), dorsum faintly, minutely punctured, bearing minute, yellowish hairs, white, recumbent, hairlike scales on the lateral surface, and long, light-brown hairs at the anterolateral margin; transverse basal groove distinct; basal half with shallow depression covered with metallic yellow ochre, round and elliptical scales; lateroventral part behind antennal scrobe densely covered with round to elliptical, white and yellow ochre scales, sparsely interspersed with short, hair-like white scales; dorsal surface weakly convex. Eyes medium-sized and feebly convex. Antenna moderately clavate, scape slightly shorter than funicle, moderately covered with fine, light-colored hairs. Funicular segments I and II almost of the same length, nearly three times longer than wide; segments III–VII slightly longer than wide; club subovoid, nearly three times longer than wide. ***Prothorax*** subglobular, as long as wide (LP/WP 1.0), faintly punctured with sparse minute hairs, widest at middle, weakly convex, with a faint groove along midline reaching the middle, and with the following scaly markings of metallic light yellow ochre, and shagreen, round scales: a) thin band at the anterior margin, b) transverse band in the entire width in middle, and c) lateroventral stripe before the coxa confluent with the anterior margin and transverse band at middle. ***Elytra*** short ovate (LE/WE 1.38), wider and longer than prothorax (WE/WP 1.67, LE/LP 2.3), black, subglabrous, strongly convex with very minute and sparse setiferous punctures, each puncture with light-colored, short seta. Elytra with scaly bands of metallic light-yellow ochre, turquoise, and blue, round scales covering stria I–IX, beginning shortly from anterior margin and extending towards apex, sometimes stria I and II interrupted at middle creating a subcircular, broad, glossy black spot without scales. Stria I–IX confluent at anterior margin; stria I, II, III, VIII, and IX confluent at the apex. Stria IV and V confluent at apical quarter. Apex with light-colored hair. ***Legs*** with moderately clavate femora. Femora covered with light-colored hair and sparse, pale-blue elliptical, hair-like scales towards apical part. Tibiae covered with subrecumbent, light-colored bristles, and weakly serrate along inner edge. Fore and mid tibiae bear a mucro at apex, and hind tibiae with apical mucrones vestigial. Tarsomeres covered with sparse pubescence. Procoxae with light-colored hair covered with pale green and light yellow-ochre round to elliptic scales on the anterior side interspersed with white hair-like scales. Mesocoxae and metacoxae with light-green hairs and sparsely covered on the anterior side with pale-blue, hairlike, round scales, less dense on metacoxae. Mesosternum covered with light-colored, adpressed bristles. Metasternum with light-colored, adpressed bristles and sparse, light-yellow ochre, round scales at lateral sides. Ventrite 1 depressed on disc with light-colored, adpressed bristles and sparse, light-yellow ochre, round scales towards lateral margin interspersed with white, hair-like scales. Ventrite 2 with long, light-brown, adpressed bristles, shorter towards margin. Ventrites 3–5 with sparse, light-colored, short bristles. Ventrite 5 flattened, apical half finely densely punctured.

***Male genitalia*** as shown in Figure 2A–C.

***Endophallus*** as shown in Figure 3. Cabras, Bollino and Medina (2018) noted that obtaining the complete eversion of the endophallus in the subgenus Orthocyrtus is particularly complicated due to the long flagellar diverticulum, and attempts are very often subject to partial failure because the flagellar diverticulum itself tends to remain invaginated. Long series of males are needed to obtain a complete or even a partial but acceptable evertion, as it was in this case (18 males available, no full, nine partial, but only five acceptable partial evertions obtained).

Even if the number of completely everted endophalluses belonging to taxa of the subgenus Orthocyrtus is still very few, it is possible to try to hypothesize the taxonomic value of this genitalic structure. From what we have observed, the flagellar diverticulum does not seem to have a species-specific morphology and is quite uniform both in shape and in length, in contrast, for example, to the subgenus Artapocyrtus (Bollino, Sandel & Yoshitake, 2019). What appears to have significant taxonomic value is the shape and presence/absence of the basal, baso-lateral, and median diverticula. In studying *Orthocyrtus*, the rate of acceptable eversion of the endophallus is about 30% of the samples tested, and although it is possible to obtain a complete evertion in approximately 1% of the samples, it will still take a long time before we can reach conclusions that are not just working hypotheses.

**Female.** Dimensions (in mm): LB 12.0–13.8 (â: 12.66), LR 2.0–2.2 (â: 2.12), WR 1.4–2.0 (â: 1.57), LP 3.2–3.8 (â: 3.56), WP 3.2–3.8 (â: 3.51), LE 8.9–10.0 (â: 9.14), WE 5.5–6.8 (â: 5.86). *N* = 15.

Habitus as shown in Figure 1D–F.

Females differ from males by the following: a) head and rostrum mostly glabrous with only a few, sparse, yellow ochre, round scales and blue, hair-like scales on lateral and latero-ventral sides, b) pronotum slightly longer than wide in female (LP/WP 1.0), c) pronotum subglobular; thin, transverse, median band interrupted at middle, d) elytra subovate (LE/WE 1.47–1.62), slightly longer and wider (WE/WP 1.72–1.79, LE/LP 2.63–2.78) than male; stria I and II interruption at middle creating a subcircular, broad, glossy black spot without scales; e) ventrite 1 flattened or slightly convex on disc. Otherwise, femail similar to the male.

#### Etymology.

The specific epithet is named after Hiraku Yoshitake (Tsukuba, Japan) for his great contribution in the advancement of studies on Pachyrhynchini in the Philippines.

#### Distribution.

Metapocyrtus (Orthocyrtus) hirakui sp. nov. is known so far only from the province of Bukidnon.

##### Brief ecological notes

**Figure 5.**
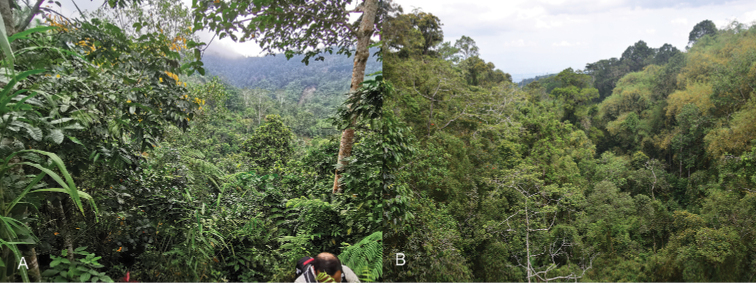
Habitat of *Orthocyrtus* spp. **A***O. davaoensis* sp. nov in Davao City **B***O. hirakui* sp. nov. in Lantapan, Bukidnon.

Specimens of M. (O.) davaoensis sp.nov. were collected on leaves of *Swietenia macrophylla* King (Meliaceae) at the ridge near Tamugan river in Calinan (Western part of Davao City) with an estimated altitude of 800 m (Fig. 5A). The biotope is a mix of secondary and agroforest. Barangay Calinan is characterized by rugged terrain and adjacent to several mountain ecosystems such as Mt. Carmen and Mt. Tamayong, with considerably higher elevation compared to the downtown area of Davao City. The river near where the new species was collected is quite pristine as evidenced by the presence of Odonates inhabiting only pristine fluvial systems such as *Eupheae amphicyana* Ris, 1930 and *Neurobasis anumariae* Hämäläinen, 1989. This biotope also has lush vegetation with several plants such as *Medinilla* sp. (Melastomataceae), *Ficus* spp. (Moraceae), *Cyathea* sp. (Cyatheaceae), and *Bambusa* spp. (Poaceae), among others. Despite the conversion of surrounding areas to maize and banana farms, it is still rich with Pachyrhynchini weevils particularly members of the genus *Metapocyrtus*. Some of the *Metapocyrtus* species documented within a radius of 500 meters from the banks of the river are Metapocyrtus (Dolichocephalocyrtus) lineaticollis Schultze, 1925, M. (Dolichocephalocyrtus) bituberosus Heller, 1912, M. (Trachycyrtus) apoensis Schultze, 1925 and M. (Trachycyrtus) adspersus Waterhouse, 1843. Compared with the aforementioned *Metapocyrtus* species which are abundant in the area, the new species is quite rare with only a few individuals documented. No species of *Pachyrhynchus* were observed. Moreover, the discovery of this new species within the remaining green spaces of Davao City reiterates the importance of our urban green spaces as a remaining haven for different species of flora and fauna and calls for immediate conservation measures.

Metapocyrtus (Orthocyrtus) hirakui sp.nov., on the contrary, was abundant in a secondary forest with some old growth trees near Lantapan, with an elevation of approximately 1200 m (Fig. 5B) The new species was present all over the area and collected on several plants, namely *Bridelia* sp. (Phyllanthaceae), *Oleandra* sp. (Oleandraceae), *Clerodendrum* sp. (Lamiaceae), *Ageratum conyzoides* (Asteraceae), *Camellia* sp. (Theaceae), *Medinilla* sp. (Melastomataceae) and *Nephrolepis* sp. (Nephrolepidaceae). However, they were more abundant along trails and open areas. As documented previously, the majority of the Pachyrhynchini including *Metapocyrtus* are often collected along trails and ridges which are either fully or partially exposed to the sun (Cabras et al. 2019). The interior of the forest does not usually give an outstanding result in terms of collecting Pachyrhynchini.

##### Notes on mimicry

Mimicry is well investigated in butterflies but far less understood in beetles which possess equally interesting mimetic patterns (Meyer 2006). The first record of mimicry among Pachyrhynchini was noted by Wallace (1889), who noticed sympatric species sharing the same integument colours and elytral patterns. This was also noted by Schultze (1923, 1925), who provided a list of 19 sympatric species of *Pachyrhynchus*, *Metapocyrtus*, and *Doliops* sharing the same coloration and patterns. He also reported 14 sympatric species of *Metapocyrtus*, exhibiting very similar elytral patterns, which were only separable after a closer inspection of diagnostic characters such as the rostrum (Schultze 1925). A study by Tseng et al. (2014) found that the diverse mimicry of Pachyrhynchini weevils are taking advantage of their coloration as aposematic signals in deterring predators, exploiting predators’ visual system. Pachyrhynchini do not possess toxins as in butterflies, but they do have a very hard elytra, which deter predators and act as a secondary defense of an aposematic insect (Schultze 1923; Wang et al. 2018). As mentioned by de Jager and Anderson (2019), mimicry can be confirmed using three conditions, namely “(1) characterizing a model, (2) identifying a receiver with a percept of said model, and (3) demonstrating that the receiver exerts selection on the mimic”. This sympatric and allopatric convergence of colors and patterns has been greatly observed among Pachyrhynchini and other Entiminae weevil groups such as *Alcidodes*, *Polycatus*, *Eupyrgops*, *Neopyrgops*, *Coptorhynchus*, and the long-horned beetle *Doliops*, which can be an outstanding example of the mimetic complex. This occurrence has also been observed among spiders, and other insects of the orders Heteroptera and Orthoptera, which show superficial resemblance and body coloration and pattern (Wallace 1889; Cabras et al. unpublished).

Metapocyrtus (Orthocyrtus) davaoensis sp. nov. was collected at nearly the same locality as *M. kitangladensis* Cabras, Medina & Zhang, 2019. This suggests a possible mimicry between these two species, for they have similar elytral markings. Photographic documentation suggests the presence of M. (O.) davaoensis sp. nov. in Marilog District, Davao City, where *M. kitangladensis* Cabras, Medina & Zhang, 2019 was also documented. The importance of the geographic distribution of the model as the limiting factor for the effectiveness of the mimicry complex has already been established. As Ries and Mullen (2008) mentioned “the advantage of mimicry does not extend beyond the range of the model”, although allopatric convergence of colors has also been documented.

Metapocyrtus (Orthocyrtus) hirakui sp. nov. belongs to a mimetic complex involving *Pachyrhynchus tikoi* Rukmane, 2016, *Doliops valainisi* Barsevskis, 2013, and *Polycatus mimicus* Bramanti, Bramanti, & Rukmane, 2020, with all species sharing a superficial resemblance. These four species were collected in an area of less than 500 m diameter and at times from the same plant. Metapocyrtus (O.) hirakui and *P. tikoi* were very abundant in the secondary forest of Lantapan, Bukidnon, and could be easily interchanged due to their uncanny resemblance. Some *Polycatus* species have previously been recorded to possibly be part of the Pachyrhynchini mimicry complex. Sometimes they exhibit a perfect mimicry, looking exactly like some Pachyrhynchini models, while at other times they exhibit imperfect mimicry and do not have exactly the same pattern. According to Forsman and Appelqvist (1998), even only the superficial resemblance and coloration of the elytra of imperfect mimicry is enough to fool visual predators which mostly rely on patterns and coloration in their choice of prey. *Polycatus* can be easily distinguished from Pachyrhynchini by the rostrum which is entirely continuous with the head, the complete metepisternal suture, the distinct squamose scutellum, the definite epistome on the rostrum, and the antenna club which has the first joint much longer than the rest, together with its basal half narrowed into a conspicuous peduncle. As expected in this case of Batesian mimicry, mimics (*Polycatus mimicus* and *Doliops valainisi*) were much less numerous in the field than models (Metapocyrtus (Orthocyrtus) hirakui sp. nov. and *Pachyrhynchus tikoi* Rukmane 2016).

**Figures 6.**
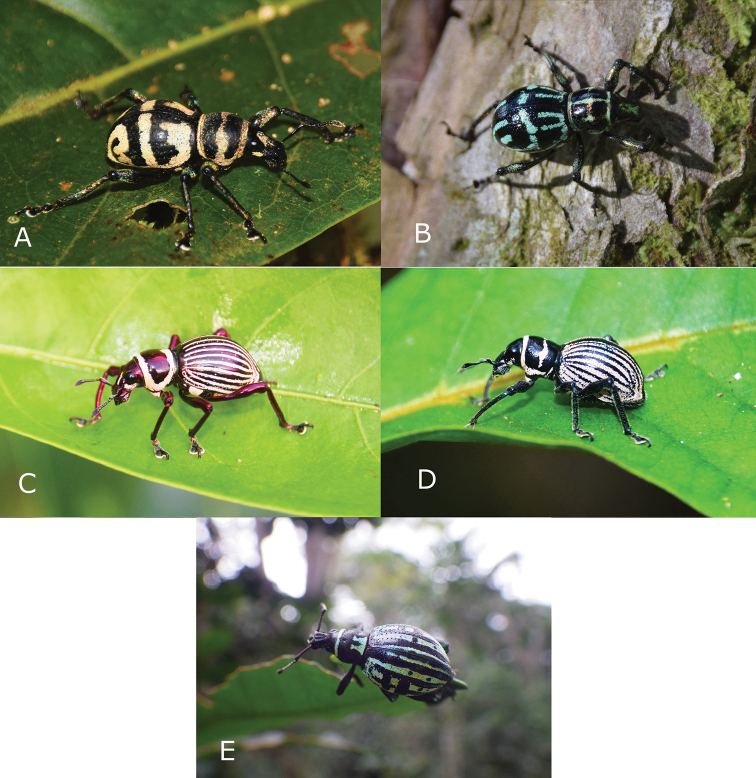
Mimicry of Metapocyrtus (Orthocyrtus) davaoensis sp. nov. and Metapocyrtus (Orthocyrtus) hirakui sp. nov. **A**M. (O.) davaoensis sp. nov. **B***Metapocyrtus kitangladensis***C***Pachyrhynchus tikoi***D**M. (O.) hirakui sp.nov. **E***Polycatus mimicus*.

## Supplementary Material

XML Treatment for Metapocyrtus (Orthocyrtus) davaoensis

XML Treatment for Metapocyrtus (Orthocyrtus) hirakui

## References

[B1] Bollino M Sandel F (2017) Two new taxa of the subgenus Artapocyrtus Heller, 1912, genus *Metapocyrtus* Heller, 1912 from the Philippines (Coleoptera, Curculionidae, Entiminae, Pachyrhynchini). Baltic Journal of Coleopterology 17(1): 1–14.

[B2] Bollino M Sandel F Yoshitake H (2019) Four new species of the genus Metapocyrtus Heller, subgenus Orthocyrtus Heller (Coleoptera, Curculionidae, Entiminae) from the Philippines. Elytra New Series 9(2): 395–340.

[B3] Bollino A Medina MN Cabras A (2020) Three new *Metapocyrtus* Heller, 1912 (Curculionidae, Entiminae, Pachyrhynchini) from Mindanao Island, Philippines. Journal of Tropical Coleopterology 1(1): 26–38.

[B4] Bontems C (2013) Le procédé Berti-Vachon d’évagination du sac interne. Nouvelle Revue d’Entomologie (Nouvelle Série) 29(1–2): 85–91.

[B5] Brown RM Diesmos AC (2001) Application of lineage-based species concepts to oceanic island frog populations: the effects of differing taxonomic philosophies on the estimation of Philippine biodiversity. Silliman Journal 42: 133–162.

[B6] Cabras A Bollino M Medina MN (2018) A new species of the subgenus Orthocyrtus, genus *Metapocyrtus* (Coleoptera, Curculionidae, Entiminae, Pachyrhynchini) from Mindanao, with notes on its ecology. Baltic Journal of Coleopterology 18(1): 39–46.

[B7] Cabras A Medina MN Zhang G (2019) *Metapocyrtus kitangladensis* sp. n., a new *Pachyrhynchus cumingii* GR Waterhouse, 1841 mimic from Mindanao Island, Philippines. ZooKeys 853: 119–129. 10.3897/zookeys.853.30595 31217720 PMC6565701

[B8] Cabras A Medina MN (2019) *Metapocyrtus ginalopezae* sp. n., a new *Orthocyrtus* from Davao de Oro, Mindanao Island. Baltic Journal Coleopterology 19(2): 205–211.

[B9] DENR Administrative Order (2019) Department of Environment and Natural Resources Administrative Order No. 2019-09. Updated National List of Threatened Philippine Fauna and their Categories. https://bmb.gov.ph/index.php/e-library/laws-and-policies/denr-administrative-orders/dao-2017-2020?download=383:denr-administrative-order-2019-09 [Accessed on 2021-3-17]

[B10] de Jager M Anderson B (2019) When is resemblance mimicry? Functional Ecology 33: 1586–1596. 10.1111/1365-2435.13346

[B11] Forsman A Appelsqvist S (1998) Visual predators impose correlational selection on prey color pattern and behavior. Behavioral Ecology 9(4): 409–413. 10.1093/beheco/9.4.409

[B12] Meyer A (2006) Repeating patterns of mimicry. PLoS Biology 4(10): e341. 10.1371/journal.pbio.0040341 PMC1617347 17048984

[B13] Ries L Mullen SP (2008) A rare model limits the distribution of its more common mimic: a twist on frequency-dependent Batesian mimicry. Evolution 62(7): 1798–1803. 10.1111/j.1558-5646.2008.00401.x 18410533

[B14] Rohling EJ Fenton M Jorissen FJ Bertrand G Ganssen G Caulet JP (1998) Magnitude of sea level lowstands of the last 500,000 years. Nature 394: 162–165. 10.1038/28134

[B15] Schultze W (1923) A monograph of the pachyrrhynchid group of the Brachyderinae, Curculionidae: part I. The genus *Pachyrrhynchus* Germar. Philippine Journal of Science 23(6): 609–673. [6 pls]

[B16] Schultze W (1925) A monograph of the pachyrrhynchid group of the Brachyderinae, Curculionidae: part III. The genera *Apocyrtidius* Heller and *Metapocyrtus* Heller. Philippine Journal of Science 26(10): 131–310. [12 pls]

[B17] Tseng HY Lin CP Hsu JY Pike DA Huang WS (2014) The functional significance of aposematic signals: geographic variation in the responses of widespread lizard predators to colourful invertebrate prey. PLoS ONE 9(3): e91777. 10.1371/journal.pone.0091777 PMC3948897 24614681

[B18] Wallace AR (1889) Darwinism: an exposition of the tTheory of Natural Selection, with some of its applications. Macmillan and Company, New York, 528 pp. 10.5962/bhl.title.17416

[B19] Wang LY Huang WS Tang HC Huang LC Lin CP (2018) Too hard to swallow: a secret secondary defence of an aposematic insect. Journal of Experimental Biology 221: jeb172486. 10.1242/jeb.172486 29180599

[B20] Yumul GP Dimalanta CB Tamayo RA Maury RC (2003) Collision, subduction and accretion events in the Philippines: a synthesis. Island Arc 12: 77–91. 10.1046/j.1440-1738.2003.00382.x

